# A Taxonomy for Research Integrity Training: Design, Conduct, and Improvements in Research Integrity Courses

**DOI:** 10.1007/s11948-022-00425-x

**Published:** 2023-04-25

**Authors:** Mariëtte van den Hoven, Tom Lindemann, Linda Zollitsch, Julia Prieß-Buchheit

**Affiliations:** 1 Amsterdam University Medical Center, Amsterdam, The Netherlands; 2 European Network of Research Ethics Committees, Bonn, Germany; 3 Zentrum für Konstruktive Erziehungswissenschaft e.V., Kiel, Germany; 4 Christian-Albrechts-University Kiel, Kiel, Germany

**Keywords:** Research integrity, Teaching and learning, Taxonomy, Evaluation, Course design, Kirkpatrick model

## Abstract

Trainers often use information from previous learning sessions to design or redesign a course. Although universities conducted numerous research integrity training in the past decades, information on what works and what does not work in research integrity training are still scattered. The latest meta-reviews offer trainers some information about effective teaching and learning activities. Yet they lack information to determine which activities are plausible for specific target groups and learning outcomes and thus do not support course design decisions in the best possible manner. This article wants to change this status quo and outlines an easy-to-use taxonomy for research integrity training based on Kirkpatrick’s four levels of evaluation to foster mutual exchange and improve research integrity course design. By describing the taxonomy for research integrity training (TRIT) in detail and outlining three European projects, their intended training effects before the project started, their learning outcomes, teaching and learning activities, and their assessment instruments, this article introduces a unified approach. This article gives practitioners references to identify didactical interrelations and impacts and (knowledge) gaps in how to (re-)design an RI course. The suggested taxonomy is easy to use and enables an increase in tailored and evidence-based (re-)designs of research integrity training.

## Introduction

For a few decades now, trainers have designed, developed and offered research integrity (RI) training to researchers of all career levels to foster responsible conduct of research (RCR). In Europe, growing interest in RI education has recently been stimulated by new and updated regulations, like the European Code of Conduct for RI by All European Academies (2017), and been furthered by projects that target the training of researchers and students, such as VIRT2UE, INTEGRITY and Path2Integrity. Even though RI education thus can build on a seemingly rich set of experiences, Kalichman (2013) observes that the answer to the question of whether RI education works is that we don’t know. One explanation for this unsatisfactory state of affairs seems that RI courses' goals, contents and approaches are often diverse (Kalichman, 2016) such that there is no common ground on what RI education is (Löfström, 2015). While there is nominal evidence of the effectiveness of RI training (Kalichman, 2016) this is often not comparable due to the variety of objectives and evaluation methodologies used. Thus, there is little evidence-based guidance on how to design and evaluate RI-courses. This contribution seeks to offer such guidance by showing how educators and curriculum designers can fruitfully apply an evaluation approach commonly used in educational research to RI trainings.

Recently, two meta-reviews analyzed the impact of training towards RI (Antes et al., 2009; Watts et al., 2017), while other meta-analyses explored the effectiveness of RCR courses qualitatively (e.g. Marušic et al., 2016; Todd et al., 2017) or took a more quantitative methods approach (e.g. Mulhearn et al., 2017). Three aspects seem to stand out in these reviews.

First, there is a massive variety of courses, learning aims, teaching and learning activities, as well as approaches that fall under the heading of RI education. Also, the perspective taken towards RI training seems to vary from prevention of falsification, fabrication and plagiarism (FFP) to a (normative) view on responsible conduct of research (Steneck, 2006). In other words, training sessions can vary from a focus on prevention and scaring people by showing misconduct and its consequences up to a focus on fostering good research practices and capacity building in students and early career researchers.

Secondly, the bulk of literature on RI courses and training often focuses on measuring the impact of these courses. It does so in the best observable ways, but that does not always necessarily align with the learning aims of the courses (Krom & van den Hoven, 2021). Steneck (2013) says that the research community still has no idea what works and what does not regarding a change in researcher behaviour. Marušic et al. (2016) describe the low quality of evidence in teaching research integrity. Löfström (2015), referencing Godecharle et al. (2013), explains that this lack of knowledge is driven by a missing consensus "about key concepts in ethics and integrity guidance (and) about content, level, timing, and frequency of ethics training and the qualifications of trainers" (p. 9).

Thirdly, meta-reviews have difficulties determining which learning activities for what target groups in what study phase are most productive in encouraging participants towards responsible conduct of research (Krom & van den Hoven, 2021). Nevertheless, there are some indications of what teaching and learning activities work concerning generalized RI learning goals (such as moral reasoning or decision-making). Watts et al. (2017) point out that "for case characteristics, the use of longer cases with moderate complexity, low affect, and low realism appear to support course effectiveness. For trainer characteristics, using multiple instructors who are experts in their professional domains also improves the effectiveness of RCR courses. Third, practice opportunities appear more beneficial when they are frequent and spaced throughout the instructional period and low in affect and realism.

Fourth, some effective delivery activities include debates, role-plays, computer simulations, and self-reflection" (p. 635). Recently, Katsarov et al. (2021) tested the robustness of findings from these meta-reviews. Eleven hypotheses were formulated and checked in the body of literature if these were robust in all studies. For example, one hypothesis was that courses offered to mono-disciplinary groups are more effective than courses offered to learners from different domains.. Contrary to expectations, Katsarov et al. (2021) found no evidence that this hypothesis holds. Nevertheless, mixed groups may be beneficial, especially for orientational learning outcomes. The authors of this study conclude that "practical course orientation with an emphasis on experiential learning and an emotional engagement with ethical decision-making appears to be the best predictor of effective RCR education" (Katsarov et al., 2021).

This unsatisfactory status quo of little sound guiding was mirrored also in the collaborative work of the three above-mentioned European RI education projects. Initial attempts to compare these trainings were hampered by two factors: First, we had no common reference points and therefore did not know what to ask for specifically and therefore repeatedly received unusable information. Secondly, we detected some gaps between intended training effects and measured effects.

To overcome this incommensurability between our three projects and to propose a more unified approach useful for the entire RI community, we introduce a taxonomy which enables informed comparisons and structured mutual learning. The taxonomy is based on Kirkpatrick's four levels of evaluation (Kirkpatrick, 1996, further developments by Barr et al., 2000, and Kirkpatrick & Kirkpatrick, 2016). Kirkpatrick's four levels of evaluation are "one of the most well-known and widely used evaluation models for training and development programs" (Reio et al., 2017, p. 35).

We build our unified approach on Kirkpatrick's model to align the design and redesign of RI training with different levels of individual, institutional and societal training effects. More precisely, the unified approach—called taxonomy of RI training—offers trainers reference points on four levels: "The levels represent a sequence or continuum of complexity. Moving from one level to the next, the evaluation process becomes more difficult and time-consuming, but it also provides increasingly more valuable information" (Reio et al., 2017, p. 36).

Unlike other approaches to design and compare RI training, such as Bloom and Krathwohl’s approach (Krathwohl, 2002), the use of Kirkpatrick's model in the taxonomy enables practitioners to concentrate on the alignment of effects inside and outside the classroom on the individual, institutional and societal level. Moreover, it gives helpful guidance on assessing these effects. Thus, the approach enables RI educators to systematically compare when and how RI training works and to adapt their courses based on sound evidence.

In this article we describe the three European projects Path2Integrity, INTEGRITY and VIRT2UE, to exemplify how the taxonomy enables mutual exchange. The unified approach also allows diversity in training while striving for high ambitions in learning outcomes and training effects. In the following, we a) describe the taxonomy for RI training in detail and b) give examples of intended training effects, learning outcomes, teaching and learning activities, and their assessment instruments from the three projects.

We propose an easy-to-use and informative four-level taxonomy for RI education that supports exchange and therefore enables better RI training design and redesign and state that enabling course designers and educators to focus on a combination of training effects and performance levels is a significant move forward in stimulating high-quality RI education.

## The Taxonomy of RI Training

**Fig. 1:**
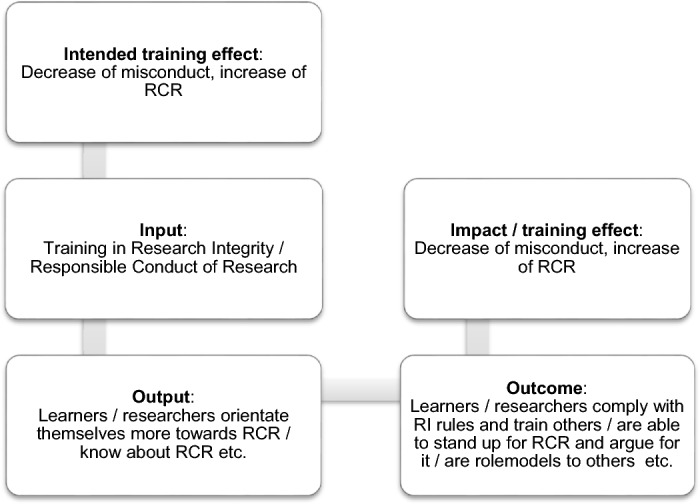
Chain of impact in RI education

While learning outcomes are unambiguous statements outlining what a learner is able to achieve as a result of completing RI training (European Commission, 2012, p. 12; Kennedy et al., 2007), learning objectives typically reflect what a trainer, program, or institution seeks to accomplish within RI training. There is a chain of impact from RI training (learning objectives) to RI learning (learning outcomes), to changed behaviour towards RI (learning outcomes), often inspired by the ambition to stimulate high-quality standards, responsible conduct of research and trustworthy science. In this article, we use the term training effects to describe the (intended) impact of RI training on different performance levels: individual, institutional, and societal (see Fig. 1).

### Students’ Performances and Training Effects from RI Training

As mentioned in the introduction, over the last decade, the research integrity community asked for more data to provide (high-quality) evidence on what type of RI training (if any) works (Steneck, 2013; Godecharle et al., 2013; Kalichman, 2013; Marušic et al., 2016; Bouter, 2020). Single studies on the efficacy and effectiveness of RI training show that training is not or only mildly effective, and some training fails to support intended training effects (e.g. Antes et al., 2010; Stephens et al., 2021; Than et al., 2020; Tarboush et al., 2020).

Furthermore, projects like VIRT2UE, Integrity and Path2Integrity show that RI training objectives can differ while sharing the same ambitions regarding training effects. For example, aims are ‘intending to build character traits such as honesty and respect’ (VIRT2UE),’stimulating empowerment’ (INTEGRITY) or to ‘conduct a dialogue on the rejection or acceptance of norms in research integrity’ (Path2Integrity). Therefore, the question how to achieve and evaluate the effects of these diverse training and facilitate exchange of information concerning their capacity to enhance research integrity is highly relevant for RI educators and course designers.

To outline and explain the diverse specifics of the three European RI education projects, in the following we apply Kirkpatricks’s model on students’ performances and training effects, showing how it can be used to structure course comparisons. Kirkpatrick’s model divides learning performances into four levels (Kirkpatrick, 1996, further developments by Barr et al., 2000; Kirkpatrick & Kirkpatrick, 2016).

**Fig. 2:**
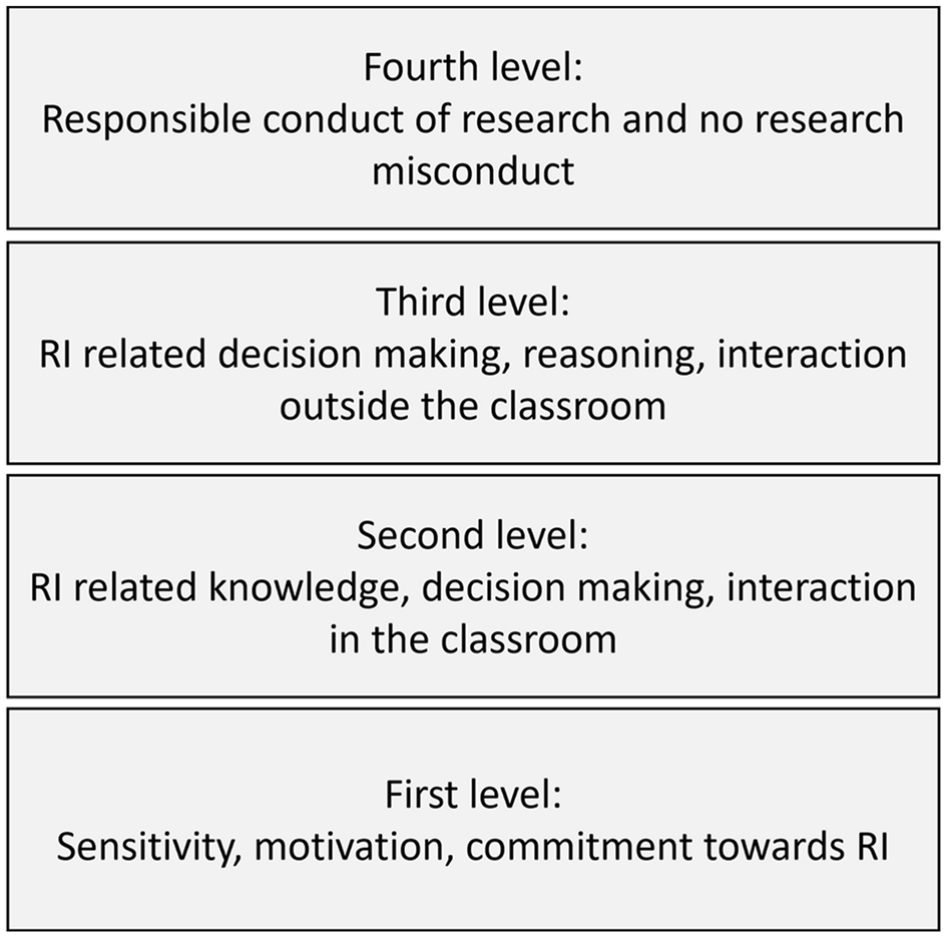
Taxonomy for RI training based on Kirkpatrick’s four levels of evaluation (TRIT)

The taxonomy of RI training (TRIT) (see Fig. 2) enables practitioners to connect the intended training effects stated at the beginning of a RI training project with the project's actual training effects (see Fig. 1). As described in Fig. 2, training effects and learners' performances of the first level relate to Kirkpatrick’s “Reaction” level, showing how favourable, engaging, and relevant RI training is for the learners. Examples for this level are:

- Path2Integrity: *I can connect the training with my everyday life.*
- INTEGRITY: *I can explain how research values are relevant to my research project.*
- VIRT2UE: *Understand the value of a dialogical attitude for group reflection.*

The second level of the TRIT relates to Kirkpatrick’s level of “Learning” and contains typical RI learning objectives. This level includes performances inside the classroom based on the learner’s RI competencies, such as reasoning, decision-making, and responsible interaction. Examples for this level are:

- Path2Integrity: *Discard arguments that cannot be justified.*
- INTEGRITY: *Transparently discuss the roles and responsibilities you and your mentors have during your Ph.D. project and how these will (have to) shift up until your graduation.*
- VIRT2UE: *Consider, choose and defend (and possibly reconsider) alternative courses of action in response to a moral dilemma in an RI case.*

Level 3 of the TRIT relates to Kirkpatrick’s level of “Behaviour” and refers to performances in which learners apply RI competencies outside the classroom. Examples for this level are:

- Path2Integrity: *Compare and prioritize different handlings of proper data management.*
- INTEGRITY: *To constructively and transparently work together with junior researchers and senior researchers.*
- VIRT2UE: *Relate virtues to norms of action when faced with moral conflicts or dilemmas.*

Level 4 of the TRIT relates to Kirkpatrick’s “Results” and includes organizational performances from learners and other people. This level includes the learners’ impact (outside the classroom) on institutions and society. For all three projects, an overlapping intended effect can be found, namely to stimulate responsible conduct of research (TRIT level 4)*.*

If evaluations ask for direct feedback about how the participants and trainers appreciate a training, the focus is on measuring effects on TRIT level 1 of the above-described taxonomy. An excellent example of such an assessment is a recent study by Abdi et al. (2021). Researchers can conduct assessments of these training effects after each learning session. TRIT level 2 of the taxonomy assesses how students perform RI competencies as a result of the training in the original training settings (see Kirkpatrick, 1996). Examples are training effects on learners’ reasoning about RI and decision-making in line with RCR in the classroom, such as those studied by Antes et al. (2007). TRITlevel 3, while also focusing on individual behaviour, investigates whether learners transfer what they have learned to settings outside the classroom. Training effects related to this level occur (or fail to occur) sometime after the actual learning session when learners face an RI problem. Examples of this level assessment are rarely found in RI literature; one notable exception is a study by Plemmons et al. (2020).

Training effects related to TRIT level 4 focus on change in research organizations and society. This article will not discuss these in detail because assessing them requires a longitudinal study capable of separating training effects from many other influences on the prevalence of research integrity. Nevertheless, the fourth level of the taxonomy reminds us that RI education should intend to impact not only what (future) researchers do in the classroom, but also how they act in and impact actual research environments (colleagues, institutions, evidence-based policy-making etc.). While an evaluation approach focused on the first three levels of the taxonomy thus cannot assess whether educational interventions enhance research integrity in the broader research environment, it is well-suited to investigate if and to what extent learners have acquired the knowledge and skills necessary to act with integrity.

The TRIT draws on yet also goes beyond constructive alignment (Biggs, 1999). It categorizes individual, institutional, and societal effects inside and outside the classroom of RI training according to the four levels of the taxonomy. In using the taxonomy, systematic knowledge can be collected, and intended training effects and impact can interconnect and pave the way towards RI training that works.

As shown in Fig. 2, such a taxonomy enables exchange between RI trainings and may lead to a more systematic approach toward effective RI training design. By concentrating on RI training effects of different depth and facets, we illustrate examples of how the three European projects can scaffold RI learning to reach their goals in the following section.

### Applying the Taxonomy for RI Training in Three European Projects

The following outlines the intended training effects of each programme, described before the respective projects started, as well as the learning outcomes, objectives and activities, how the projects administered them, and the evaluation instruments developed to assess the training programmes. It is important to note that none of the three programmes was planned based on TRIT, and that applying TRIT demonstrates a lack of direct connections between intended training effects and their assessment (see Table 2).

All three projects ultimately aim to foster a culture of research integrity (TRIT level 4) based on the core principles of reliability, honesty, respect, and accountability. However, their programmatic approaches and normative conceptualizations of research integrity differ, affecting their learning objectives. Path2Integrity uses a discourse ethics approach to research integrity that emphasizes the value of dialogues about norms. In contrast, INTEGRITY aims to empower learners and thus focuses on building capacities that help overcome structural obstacles to acting with integrity. VIRT2UE takes a virtue ethics approach and seeks to support learners in cultivating character traits conducive to research integrity by reflecting on questions about who they wish to be and the action implications following from this reflection.

### Path2Integrity

#### Intended Training Effects

Applying the taxonomy on intended training effects, we observe that the overall training effect of Path2Integrity intends to provide a positive culture of research integrity, which relates to TRIT level 4. At TRIT’s level three, the training wants to increase “research integrity knowledge and research integrity reasoning” (Priess-Buchheit et al., 2020, p. 6) to open a door into the scientific community and engage participants in a dialogue about research integrity. The Path2Integrity programme promotes learners “to conduct a dialogue on the rejection or acceptance of norms in research integrity” (Priess-Buchheit et al., 2020, p. 23), level two of the taxonomy, to reach these training effects.

Path2Integrity offers three handbooks of instructions and 27 so-called learning cards. These learning sessions are called the Path2Integrity Learning Card Program (P2ILC). The programme was developed in 2019 and 2020 (Hermeking & Priess-Buchheit, 2022; Priess-Buchheit, 2020).

The centre of this programme is a dialogical approach, which can be described as the opposite of debate (Widdershoven & Solbakk, 2019). The P2ILC programme enables each participant to rationally lay out their position on good scientific practice as well as the ways in which one would explain and justify their position to others. As opposed to debate, participants are encouraged to build sound arguments by listening actively and (if necessary) countering good arguments. (Priess-Buchheit, 2020, p. 55)

#### Learning Objectives and Outcomes

**Fig. 3:**
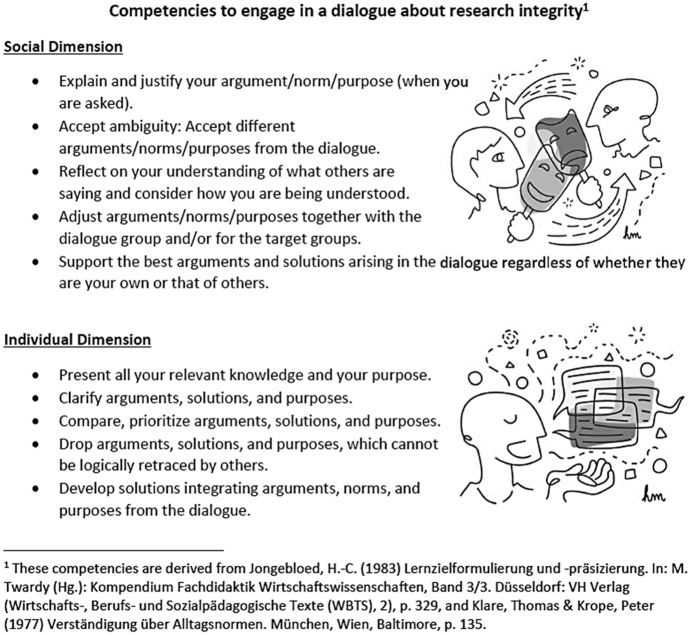
Competencies to engage in a dialogue about research integrity

As seen in Fig. 3, the project distinguished learners’ individual and social activities to consider the social dimension of different teaching and learning activities. By transferring and adjusting each learning objective to three different target groups (upper-level high school students, university students, and early career researchers), Path2Integrity shaped performance-based learning outcomes, which are targeted in the twenty-seven P2ILC sessions.

One card (Priess-Buchheit, 2021a) for master students and early career researchers, for example, supports *clarifying arguments, solutions, and purposes in RI-related interactions* and outlines, that learners *describe the values of a researcher* (TRIT level 2). Path2Integrity also thrives on instructing learners (Priess-Buchheit, 2021b) *to explain and justify their argument, norm, and purpose (when they are asked)*, by expecting them to “*refer to codes and regulations*” (TRIT level 2). Another learning objective on a more complex level (Lindemann & Priess-Buchheit, 2021) is *to develop solutions by integrating arguments, norms, and purposes from the dialogue*. To "*adjust research procedures, if necessary*” or to “*discard arguments that cannot be justified*” (TRIT level 2) lead learners toward this goal.

Path2Integrity uses three main learning activities to achieve its above-described learning outcomes. By engaging participants in (a) role-play, (b) storytelling, and (c) coming to agreements, students outline not only their knowledge about research integrity but also discuss their roles (awareness/sensitivity, reasoning, and commitment) regarding reliable research.

These collaborative learning methods support Path2Integrity’s dialogical approach. “Vivid storytelling and … role-play enable students, (under)graduates and young researchers to acknowledge conflicting purposes, power structures, (sub‑)cultural habits and knowledge. They also lead them to rationally … [lay out their] research integrity, listen to statements of others … and to be ready to outline their knowledge about research integrity" (Priess-Buchheit et al., 2020, p. 20). Those three Path2Integrity learning and teaching activities mentioned above follow the dialogical principle and aim to build a common language, answer questions, or develop solutions for a (shared) purpose. They should be conducted under the condition that "equal rights and equal duties are to be demanded of all participants. Neither authority nor violence, deception nor irreconcilable promises should occur therein" (Janich, 2009, pp. 20–21, translated by one of the authors).

Path2Integrity's activities are in line with the findings of ethics and research integrity experts (Grose-Fifer, 2017; Löfström, 2016; McWilliams & Nahavandi, 2006; Poling & Hupp, 2009; Poorman, 2002), who state that students engaging in role-playing are more aware of the complexities of ethics, increase their critical and reflexive thinking, apply concepts, get emotionally engaged, and take over personal accountability.

In the project's first year, Path2Integrity added *Coming to an Agreement* as a third main learning activity in order “to create learning situations in which participants focused on reaching a common decision—without being distracted” (Priess-Buchheit, 2020, p. 56). Trainers apply the three main learning activities in different tasks appropriate to each target group. Path2Integrity offers its learning sessions as printed learning cards for onsite classroom teaching and a free-to-use learning management system, which allows trainers to facilitate these collaborative activities in an online setting.

#### Assessment

Path2Integrity evaluates its RI training via a mixed-methods approach by (a) collecting feedback from participants and trainers (Zollitsch & Wilder, 2022; Zollitsch et al., 2020), (b) conducting group discussions with participants as well as c) recording quantitative data in a pre-post-test design.

The feedback sheet for the participants asks how the participants feel about the training and their learning experience. In addition, the feedback sheet contains questions regarding the social climate within the group, the trainer's competence, the participation possibilities, the overall satisfaction with the training, and the personal relevance (TRIT level 1). Likewise, the trainer's feedback gives insights into what worked well and what obstacles occurred from the trainer’s point of view (TRIT level 1).

The P2I questionnaire (Zollitsch et al., 2022) does not focus on dilemmas or ethical questions but instead asks participants to suggest research practices and justify these practices. This questionnaire follows a four-tier test design (first implemented by Treagust, 1988), records quantitative data, and gives insights into how learners argue in favour of RI (TRIT level 2).

The group discussions give insights into long-term changes in how participants behave after the training. They are conducted at least 2 weeks after the training. The behaviour is analyzed with the documentary method (Bohnsack et al., 2010), aiming to reconstruct the implicit knowledge underlying everyday practice. Results are TRIT’s level 3 and outline habitualized actions independent of individual intentions and motives (Bohnsack et al., 2010).

Path2Integrity’s learning outcomes, activities, and assessment pursue the training effects and concentrate on a dialogical structure of RI. The described assessment concentrates on levels one to three and does not cover level four, the culture of research integrity. Path2Integrity's results show that the programme's learning activities can increase research integrity knowledge and research integrity reasoning and lead students to conduct a dialogue on the rejection or acceptance of norms in research integrity (Hermeking & Prieß-Buchheit, forthcoming; Hermeking & Priess-Buchheit, 2022; Priess-Buchheit, 2020).

### INTEGRITY

#### Intended Training Effects

The overall intended effect of the INTEGRITY project is to stimulate *empowerment* toward responsible conduct of research (TRIT level 4) so that learners can anticipate what research integrity will entail in the future. The notion of empowerment has been utilized based on writings on empowerment, starting with Paulo Freire's *Pedagogy of the Oppressed* (Freire, 1970). The main idea is that empowerment requires (1) capacity building of students at all relevant levels targeted in the project (upper-level high school students, undergraduate students and early career researchers) and (2) that innovative and appealing educational tools need to be developed in an evidence-based manner. Therefore, empirical studies and literature review supported the intended training effect, which served as input for the teaching philosophy and competency profile.

The project defined the following main characteristics that shape the idea of what empowerment towards RCR entails as a normative concept: Empowerment is (1) about building capacities of individual researchers, who function in institutional and systemic contexts of research practices; (2) about teaching students to take control; (3) about learning to develop critical autonomy and (4) about stimulating an attitude of openness, a 'feeling up to' and courage to address issues of integrity in practice. These intended effects are mainly at TRIT’S level 3 and level 4.

#### Learning Objectives and Outcomes

The general view on empowerment has been developed into a competence profile (see Table 1) which describes basic competencies for all target group students with some additional competencies. The following shows how trainers can interpret competencies differently in various study phases. Competence '*is able to apply rules of responsible conduct of research and research/academic values to one's project/field and to conduct one's research (project) according to RCR standards, and values* (TRIT level 3) can be interpreted in a high school context in learning about the relevant rules and regulations, e.g. use of literature (referencing) and how to apply this in one's written work, while for a Ph.D. or postdoc this competence can be used to help them learn more about the relevance of data management and how to apply this in their project. The competencies have been used to define learning objectives, assess learning outcomes, and apply to various levels in TRIT.

**Table 1:** Competence profile

A good researcher …
Has basic knowledge on what (a) research (project) entails (research cycle, designing a study, using appropriate methodology, collecting & analyzing data, reporting findings) and what challenges this brings with it;
Can explain rules and regulations regarding academic & research integrity (like codes of conduct, rules on plagiarism, etc.) and apply them to generic cases
Is able to apply rules and regulations of responsible conduct of research and research/academic values to one’s own project/field, and to conduct one’s research (project) according to RCR standards and values;
Can recognize and point out what integrity issues are relevant in one’s own context and how they relate to debates on Responsible Conduct of Research (RCR);
Is able to identify and reflect on relevant RCR aspects in a given situation;
Is able to determine relevant strategies in a situation in which RCR is at stake;
Can determine an appropriate course of action in a situation in which integrity is at stake (also in consultation with others);
Is an active bystander (i.e. takes active responsibility) when encountering situations that could jeopardize RCR;
Expresses adherence to norms of responsible conduct of research;
Demonstrates in one’s reflections and decisions that one feels up to addressing issues of RCR and integrity with others;
Recognizes, and is able to withstand stimuli to condone misconduct;
Understands the institutional context of integrity issues, and how one’s individual role is sometimes limited yet relevant;
Acts respectfully towards others (humans, animals, nature) when conducting research (projects)
Acts with honesty, responsibility, and transparency as core values of research;
Demonstrates sufficient analytic, problem-solving, and communicative skills in discussions and deliberations on RCR issues

Some examples of how the competencies have been used to define learning objectives in courses aimed at Ph.D. students:

- *After this e-course, I can explain how research values are relevant to my research project. (TRIT level 2)*
- *I understand how specific values are underlying regulations regarding data management and ethics review. (level 2)*
- *I can distinguish the relevant aspects of a data management plan. (level 3)*

Regarding the undergraduate students, the project developed an interactive website (www.integgame.eu) where students go through a narrative which presents several real cases for undergraduate students. The consequences of choices that they must make during the narrative will be shown. Students can access these Integrity Games individually: Students do quizzes, play the narrative and get background information on integrity issues. The tool is complementary to classroom teaching to allow discussion among students about their own experiences with the topic, covering three general themes: 1. Drawing on the work of others (including plagiarism) 2. Collaboration 3. Collecting, analyzing and presenting data. (See for more information: d.4.8 Prototype tools for bachelor students).

The aim is to 'spark interest, reflection and learning, which relate primarily to TRIT’s levels 1 and 2. The project develops three small private online courses (SPOCs) for Ph.D. students that focus on specific topics related to integrity (namely, RCR in supervision and mentoring relationships, the role of data in research integrity issues and reviewing, and authorship). Students study relatively independently from a teacher in small private online courses. The courses offer a mixture of independent study, active assignments and group interaction. The specific assignments and activities designed are oriented toward creating an active awareness (TRIT level 2), learning reflective skills (TRIT level 2) and stimulating a proactive attitude (TRIT level 2). For example, in the supervision and mentoring course, participants are explicitly instructed to organize a meeting with their supervisor to talk about the topics relevant to them in the course. At the end of the course, they are asked to report on this meeting. This assignment helps participants to apply relevant integrity topics in their context outside the classroom.

#### Assessment

Integrity evaluates the tools in different ways. For the undergraduate tool, a randomised controlled trial was conducted with students from Denmark and Hungary, using a pre-and post-test design with a timetable. The assessment focuses on increasing the understanding of integrity issues and if students develop a higher motivation to behave responsibly. This aligns much with TRIT level 2.

Regarding the Ph.D. course evaluation, a mixed-method approach is used to see if students’ competencies increase. Data are collected in three different ways: first, there is a pre-and post-survey, using the validated Professional Decision-making in Research (PDR tool) (developed by the Bioethics Research Center in St Louis), in which cases are presented to respondents with multiple choice answer options. This test evaluates at’TRIT’s second level and has two forms (A and B) to take pre-and post-intervention. Also, in-course data (assignments) are evaluated to see what level of reasoning skills learners possess (TRIT level 2). In reflection questions, learners are asked about progress in their competencies, also related to becoming more proactive on integrity issues in their work (TRIT level 3).

### VIRT2UE

#### Intended Training Effects

VIRT2UE is a train-the-trainer programme that addresses all researchers and educators who want to become RI trainers or broaden their skillset. The overall aim of the VIRT2UE project is to foster reflection on and training in the character traits of researchers themselves. The overarching learning objective of the programme is to foster RI (TRIT level 4) by enabling participants to design and teach learner-centred RI courses for researchers of all career levels (TRIT level 3). The training takes a virtue ethics approach to RI, and it thus focuses on moral character development and the cultivation of habits conducive to acting with integrity. To bridge theory and practice, trainers learn how to foster reflection on intellectual and moral virtues relevant to research and apply them to cases and experiences. These are typical TRIT level 4 ambitions.

#### Learning Objectives and Outcomes

The specific learning objectives of the VIRT2UE programme can be differentiated into objectives for trainers in training and outcomes for researchers trained by trainers. In this article we focus on the former.

Learning objectives for trainers cover several levels in TRIT:

- *Recognize and formulate a moral dilemma in an RI case (TRIT level 2).*
- *Recognize whether a group discussion reflects dialogue or debate features (TRIT level 2).*
- *Facilitate a dialogue among participants to foster reflection and ethical deliberation processes in their training (TRIT level 3).*

As these learning objectives indicate, the focus of the VIRT2UE programme is on connecting theory to practice, emphasizing the latter. Learning outcomes focus on applying virtues to RI cases rather than on teaching ethical theory to reflect the aim to support researchers in acting with integrity. How researchers can apply virtues to cases and how trainers can foster moral reflection in others is the focus of the synchronous, in-person component of the VIRT2UE programme, which consists of five exercises.

Three online courses precede the in-person part of the programme. The first course asks learners to apply the principles of RI to their context. The second course highlights the relevance of virtue ethics for RI and invites learners to self-assess their knowledge, relate to and apply introduced concepts in reflexive exercises, and reflect on their relevance to their daily research practices. Finally, the third course challenges learners to reflect on the current research culture and conditions that undermine values and incentivize the development of vices. Drawing on the knowledge learners have acquired in the online courses, the in-person exercises use a combination of experiential learning (e.g. experiencing a dialogue in contrast to a debate, game-based deliberations) and collaborative learning (e.g. small group and plenary discussions) and interactive instruction (group assignments).

#### Assessment

VIRT2UE assesses whether learning objectives have been met differently, utilizing both self-assessments and formative assessments. The taxonomy provides a helpful framework to illustrate how components and effects of VIRT2UE are evaluated throughout the programme.

Upon completion of the whole course, participants are asked to fill out an evaluation survey asking about their impressions, what they liked, what they disliked (TRIT level 1), whether they feel competent to organize their own RI course and numerous other questions (TRIT level 3). Self-assessments related to the online courses refer to first and second-level performances because these courses focus on raising moral sensitivity, conveying knowledge about RI and virtue ethics, and seek to facilitate applications of this knowledge to concrete situations.

The self-assessments are intended to enable learners to obtain feedback on whether they have accomplished the learning objectives and to make an informed judgment about whether they need to rehearse some contents. Trainers ask learners to fill out reflection forms after using the exercises in their training to gather tentative information on effects on the third level. The learners describe what went well and what went less well.

## Reflection and Outcome

As outlined in the beginning, first attempts to learn from each other in the projects failed due to diverse approaches and missing reference points. To overcome this challenge and to utilize experiences we suggested to introduce the TRIT. As can be seen in Table 2 the TRIt enables an exchange between the RI education projects and support a systematic overview and effective (re-)design in the future.

Therefore, the taxonomy for RI training (TRIT) which is based on Kirkpatrick’s evaluation model enhances dialogue and knowledge-sharing and increases the quality of RI training.

We have used three recent EU projects to exemplify this approach. All three projects share high ambitions that locates their intended training effects at TRIT’s level 4. Even if they all ultimately aim to stimulate responsible conduct of research and reduce research misconduct, they pursue this aim in very different ways (see Table 2). However, they continue to face an information deficit when trying to connect their assessments to intended effects in behavioural change, even though all three use a more or less elaborate evaluation scheme (see the comparison between target and index/test instrument on level 4 for all projects in Table 2). Notwithstanding this notable problem, all three projects contribute to improving the evidence on how RI can be taught effectively by developing initial schemes and examples of how trainers can assess the effects of RI training systematically on TRIT’S first three levels.

**Table 2:** Projects’ aims on TRIT levels

	Path2Integrity		Integrity		VIRTUE	
	Target	Index/test-instrument	Target	Index/test-instrument	Target	Index/test-instrument
Projects’ aim on level 4	RCR	None exists	RCR	None exists	RCR	None exists
Projects’ examples for level 3	Compare and prioritize different handlings of proper data management	P2I group discussions	Constructively and transparently work together with junior researchers and senior researchers	Integrity reflection questions	Relate virtues to norms of action when faced with moral conflicts or dilemmas	VIRTUE evaluation survey
Projects’ examples level 2	Discard arguments that cannot be justified	P2I pre-post questionnaire	Transparently discuss the roles and responsibilities you and your mentors have during your Ph.D. project and how these will (have to) shift up until your graduation	(1) Integrity randomised control trial (2) PDR from St. Louis (3) assignment evaluation	Consider, choose and defend (and possibly reconsider) alternative courses of action in response to a moral dilemma in an RI case	VIRTUE self-assessment
Projects’ examples level 1	None exists	P2I feedback sheet	None exists	Evaluation module using likert scales and open questions	None exists	VIRTUE self-assessment

All three projects use direct learners’ feedback to collect information about learners’ reactions. All three projects also refer to TRIT’s second level (see Table 2), albeit based on different assessment procedures. Path2Integrity uses a questionnaire asking participants to suggest and justify a scientific practice. INTEGRITY asks how specific competencies are stimulated, and VIRT2UE uses self-assessments and non-recorded classroom talks to evaluate their outcomes.

Also, all three projects assess whether participants in training do well concerning the second level of Kirkpatrick's model by informally evaluating whether classroom activities (e.g., the argumentative quality and style of discussions) align with the stated learning objectives. Some exercises, for example, include plenary and group discussions that involve sharing arguments, results and insights and provide learning opportunities for trainers by asking questions. These feedback mechanisms facilitate adjusting learning activities, if necessary. Forthcoming publications of the projects will give more information on how effective each program at each level is.

Moreover, TRIT underscores the relevance of constructive alignment in courses (cf. constructive alignment: Biggs, 1999) and emphasizes outcomes-based (Biggs & Tang, 2020) and impacts-based designs. In a nutshell, if a trainer formulates learning objectives only at level 2, it does not make sense to expect learners to perform well on level 3 when assessing a training (see Reio et al., 2017, p. 37). Also, if the ultimate intended training effect is a typical level 4 or 3 ambition, course aims, materials, and working methods should relate closely to these aims. Therefore, using TRIT in planning and exchange can highlight inconsistencies between levels at the stage of objectives, outcomes, and assessment, and emphasizes the need for sound pedagogical theory and evaluation designs that trainers use widely in educational research. Overall, trainers can use the taxonomy retrospectively, as it can reveal them blind spots in their evaluations and what could be improved. It can also be used prospectively, stimulating to align RI courses better with intended outcomes (Krom & van den Hoven, 2021).

A clear advantage of TRIT is that it also shows that there does not have to be a 'one size fits all' approach to RI training. The three EU projects have different teaching philosophies, while sharing the same intended training effect and use different working methods and learning objectives to achieve these. Despite differences in their conceptualizations of RI, Path2Integrity, INTEGRITY and VIRT2UE walk on shared grounds concerning the chain of impact (Fig. 1). Thus, we advocate in favour of a more unified approach to course design and evaluation that enables structured mutual learning. In this way, we depart from many current generalizations in RI education research, which, in our view, often hamper efforts to identify specific RI training designs and redesigns by unduly focusing on identifying a single best approach to RI education—rather than on how trainers should design and assess courses. Shifting the focus to course design and assessment implies that RI educators need not only content knowledge about RI but also ample pedagogical knowledge and content knowledge because reliance on a single best approach to RI education across all groups of learners and scientific disciplines is in all likelihood impossible. Instead, learning objectives and outcomes, as well as course activities, need to be developed, assessed, and adapted based on learners' needs and existing competencies.

In shedding light on the projects' learning objectives and outcomes, teaching and learning activities, and assessments, we want to facilitate constructive exchange between courses and training programs based on detailed and systematic descriptions of the interrelations and influences between different training components concerning their intended and actual effects. Thereby we aim to push for more evidence-based RI education. As shown in the sections above, mapping and measuring training components based on a sound evaluation framework that has proven useful in educational research helps clarifying the type of information needed to foster RI on the individual, institutional and societal level. By comparing different RI trainings, practitioners can obtain essential information and critically reflect on how they can improve future RI education. We illustrated that comparisons of RI training based on the proposed TRIT enable RI trainers and course designers to collect information conducive to supporting improvements in future RI trainings.

Therefore, we recommend using the TRIT to compare RI trainings and identify didactical interrelations and impacts and (knowledge) gaps in how to design RI courses. TRIT is easy to use, overcomes the oversimplification of learning objectives in recent meta-reviews, and enables comparison and (re-)design of RI training. Further research needs to validate whether the taxonomy will be a helpful tool in systematizing RI training and whether it can sufficiently support systematic RI training design and redesign endeavours.

## Data Availability

Readers can find the learning objectives on the respective project's websites. www.h2020integrity.eu. www.embassy.science/training.eu. www.path2integrity.eu
